# Are Sirtuins 1 and 2 Relevant Players in Relapsing–Remitting Multiple Sclerosis?

**DOI:** 10.3390/biomedicines12092027

**Published:** 2024-09-05

**Authors:** Justyna Chojdak-Łukasiewicz, Anna Bizoń, Aleksandra Kołtuniuk, Marta Waliszewska-Prosół, Sławomir Budrewicz, Agnieszka Piwowar, Anna Pokryszko-Dragan

**Affiliations:** 1 Clinical Department of Neurology, Faculty of Medicine, Wroclaw Medical University, Borowska 213, 50-556 Wroclaw, Poland; marta.waliszewska-prosol@umw.edu.pl (M.W.-P.); slawomir.budrewicz@umw.edu.pl (S.B.); anna.pokryszko-dragan@umw.edu.pl (A.P.-D.); 2 Department of Toxicology, Faculty of Pharmacy, Wroclaw Medical University, Borowska 211, 50-556 Wroclaw, Polandagnieszka.piwowar@umw.edu.pl (A.P.); 3 Department of Nursing and Obstetrics, Faculty of Health Sciences, Wroclaw Medical University, Bartla 5, 51-618 Wroclaw, Poland; aleksandra.koltuniuk@umw.edu.pl

**Keywords:** sirtuins, SIRT1, SIRT2, multiple sclerosis, disability, biomarkers

## Abstract

SIRTs were demonstrated to play an important role in inflammatory, degenerative, and metabolic alterations, constituting the background of the central nervous system. Thus, they seem to be an appropriate object of investigation (as potential biomarkers of disease activity and/or novel therapeutic targets) in multiple sclerosis (MS), which has a complex etiology that comprises a cross-talk between all these processes. The aim of this study was to evaluate the levels of SIRT1 and SIRT2 in the serum of patients with the relapsing–remitting type of MS (RRMS), as well as their relationships with various aspects of MS-related disability. Methods: A total of 115 patients with RRMS (78 women, 37 men, mean age 43 ± 9.9) and 39 healthy controls were included in the study. SIRT1 and SIRT2 were detected in the serum using the enzyme-linked immunoassay (ELISA) method. In the RRMS group, relationships were investigated between the SIRT 1 and 2 levels and the demographic data, MS-related clinical variables, and the results of tests evaluating fatigue, sleep problems, cognitive performance, autonomic dysfunction, and depression. Results: The levels of SIRT1 and SIRT2 in RRMS patients were significantly lower than in the controls (11.14 vs. 14. 23, *p* = 0.04; 8.62 vs. 14.2, *p* < 0.01). In the RRMS group, the level of both SIRTs was higher in men than in women (15.7 vs. 9.0; 11.3 vs. 7.3, *p* = 0.002) and showed a significant correlation with the degree of disability (R = −0.25, *p* = 0.018). No other relationships were found between SIRT levels and the analyzed data. Conclusions: The serum levels of SIRT1 and 2 were decreased in the RRMS patients (especially in the female ones) and correlated with the degree of neurological deficit. The role of SIRTs as biomarkers of disease activity or mediators relevant for “invisible disability” in MS warrants further investigation.

## 1. Introduction

Silent information regulators—sirtuins (SIRTs)—are histone deacetylases, a family of enzymes that are responsible for posttranslational modifications of proteins and involved in multiple metabolic, energetic, and proliferation cellular processes to maintain cell homeostasis despite adverse external factors [1,2]. Due to their multidimensional activity, SIRTs have recently been recognized as possible relevant players in the pathomechanisms of various human diseases, including those affecting the nervous system. SIRTs are widely expressed in the nervous tissue and engaged in processes of maturation, viability, and death [3,4]. Within the central nervous system (CNS), they are supposed to be involved in advanced functions of neuronal networks that are associated with cognition, behavior, and mood. SIRTs were demonstrated to play an important role in inflammatory, degenerative, and metabolic alterations, constituting the background of CNS disorders [5]. Thus, they seem to be appropriate objects of investigation in multiple sclerosis (MS), which has a complex etiology that comprises a cross-talk between all these processes.

MS is a chronic CNS disease, which causes multifocal lesions (including damage to the myelin sheath and loss of axons) throughout the brain and spinal cord. They result in various signs of neurological deficit, which occur with changing dynamics in the course of the disease and contribute to accumulating disability. In the most frequent, relapsing–remitting type of MS (RRMS), new or recurrent symptoms appear during acute exacerbations and become completely or partially resolved, followed by periods of a stable condition. A gradual worsening of symptoms is observed in the primary or secondary progressive types (PPMS, SPMS). According to the more recent classification, each of these main MS phenotypes (relapsing or progressive) can also be modified by the temporal presence of disease activity and progression [6,7]. Apart from the most common symptoms of MS (impairment of vision, gait, or balance and sensory or bladder dysfunction), increasing attention has recently been paid to conditions like fatigue, cognitive decline, mood, and sleep disturbances, which are difficult to capture (thus, they are called “invisible disability”) and yet cause a serious burden to the patients [8]. Despite substantial progress in the knowledge about the MS background (immune dysregulation accompanied by neurodegeneration, resulting from interacting genetic and environmental factors), as well as diagnostic and therapeutic opportunities, some aspects of the disease still need to be elucidated and addressed. Thus, there is an ongoing search for potential monitoring and predictive biomarkers of the disease activity and/or novel therapeutic targets.

Among SIRTs, SIRT 1 and 2 seemed most promising for these directions of investigation in the field of MS. SIRT 2 plays an important role in the formation and function of the myelin sheath, while SIRT 1 contributes to the development and maturation of neurons and synapses. They both were shown to be engaged in substantial signaling pathways relevant to the modulation of inflammatory and degenerative processes in the CNS and the links between them [1,2].

In view of that, SIRT1 and SIRT2 have been investigated in some experimental models of MS and less frequently in clinical studies [6,7,8]. The findings from these studies seem promising but inconclusive and encouraged further exploration. Moreover, evidence from studies on SIRTs in other CNS disorders suggests that their putative role in the background of components of MS-related “invisible” disability, which has not been investigated so far, also deserves attention [9,10].

The aim of this study was to evaluate the levels of SIRT1 and SIRT2 in the serum of patients with the relapsing–remitting type of MS (RRMS), as well as their relationships with various aspects of MS-related disability.

## 2. Materials and Methods

### 2.1. Study Design and Participants

The participants in the study were recruited from the patients diagnosed with relapsing–remitting MS (RRMS) according to the current version of McDonald’s diagnostic criteria [7]; the patients were undergoing regular follow-up for at least 6 months at the Department of Neurology, Wroclaw Medical University, hospitalized, or consulted between January and April 2022. The exclusion criteria included relapse and treatment with corticosteroids within the preceding 3 months, starting or switching disease-modifying treatment (DMT) within the preceding 3 months, active or chronic infection or other coexisting inflammatory condition, use of antibiotics within the preceding month, history of alcohol or drug abuse. Finally, the study group comprised 115 patients (78 women and 37 men, aged 43 ± 9.91). The control group consisted of 39 healthy volunteers, matched for age and gender to the study group.

### 2.2. Clinical Assessment

#### 2.2.1. Clinical Parameters

Body weight, height, and body mass index (BMI) were determined in all participants. In the study group, the data concerning the course of MS (including duration of the disease, DMT used, and current degree of disability assessed using the Expanded Disability Status Scale—EDSS [11]) were collected from medical charts.

The following self-assessment standardized questionnaires were completed by all the participants:

1. Athens Insomnia Scale (AIS) [12] and Insomnia Severity Index (ISI) [13] to assess sleep problems.
2. Fatigue Severity Scale (FSS) [14] and Modified Fatigue Impact Scale (MFSI) [15] to evaluate the level of fatigue.
3. Symbol Digit Modalities Test (SDMT) [16] as a measure of cognitive function.
4. Composite Autonomic Scoring Scale for Laboratory Quantification of Generalized Autonomic Failure (Low questionnaire)—to assess the symptoms of autonomic dysfunction [17].
5. Hospital Anxiety and Depression Scale (Bjelland et al. 2002) to detect mood or anxiety disorders [18].

#### 2.2.2. Biochemical Parameters

From all the patients, approximately 15 mL of peripheral venous blood was collected using tubes containing a clotting activator (BD Vacutainer™ Serum Tubes, Cat. No.: SKU: 367815, BD, Herlev, Denmark). The samples were immediately centrifuged at 2500× *g*/15 min., and the serum was separated. The serum was immediately frozen at −80 °C until use. The concentration of SIRT1 and SIRT2 was determined using the enzyme-linked immunoassay (ELISA) method. The Human Sirtuin 1 ELISA Kit (ref. No. E2557Hu, Bioassay Technology Laboratory, Shanghai Korain Biotech Co., Ltd., Shanghai, China) and the Human Sirtuin 2 ELISA Kit (ref. No E2558Hu, Bioassay Technology Laboratory, Shanghai Korain Biotech Co., Ltd., Shanghai, China) were employed for this purpose. To measure the SIRT1 or SIRT2 levels, standard curves ranging from 0.2 to 60 mg/mL were prepared according to the manufacturer’s instructions. Briefly: serum samples and SIRT1 or SIRT2 standards were added to a 96-well plate. Biotin-conjugated anti-SIRT1 or SIRT2 antibody was pipetted into the wells containing serum samples, and streptavidin-horseradish peroxidase was added to each well. The plate was mixed, covered, and incubated for 60 min at 37 °C. Following incubation, the wells were washed five times, and substrate solution was added. After 10 min at 37 °C, the color development was inhibited by adding stop solution. The absorbance was then measured at λ = 450 nm using a microplate reader (Synergy HTX, Biotek, Shoreline, WA, USA).

Collection of blood samples and the clinical assessments were performed during the same session. Considering potential circadian fluctuations of the SIRT levels, as well as the patient’s condition (especially in terms of fatigue or alertness), these sessions were planned at the same time of the day for all the study participants (in the morning hours—the most convenient for optimal assistance for the patients).

All the participants were fully informed about the aim and course of the study and provided written consent prior to their inclusion. The study was conducted according to the guidelines of the Declaration of Helsinki and Good Clinical Practice. The project of the study was approved by the Bioethics Committee at Wroclaw Medical University (decision number KB-148/2022).

The serum levels of SIRT 1 and SIRT2 were compared between the RRMS patients and the control group. In both groups, the relationships were analyzed between SIRT level and demographics and, in the RRMS group, the disease duration, EDSS score, and the results of AIS, ISI, FSS, MFIS, SDMT, HADS, and the Low questionnaire.

### 2.3. Statistical Analysis

All data analyses were performed using R (version 4.0.2, R Foundation for Statistical Computing, Vienna, Austria). Descriptive statistics were used for the number of cases, percentages, and mean ± SD. The data were reported as mean values with standard deviations. The normality of distribution for all the continuous variables was verified with a Shapiro–Wilk test, and variance homogeneity was checked with the F test to select appropriate statistical methods. If both assumptions were met, the Student t-test was applied to compare serum SIRT1 and SIRT 2 levels between the study groups. In the case of normally distributed variables that did not comply with the variance homogeneity condition, the Welch *t*-test was applied. Otherwise, a Mann–Whitney U test was used. For correlation analysis, the assumption of the normally distributed variables was also checked. In the case of normally distributed variables, a Pearson correlation was used to assess the clinical–radiological correlations. Otherwise, and in the case of non-linear dependencies, a Spearman correlation was applied. Differences were considered statistically significant when *p* < 0.05.

## 3. Results

The demographic and clinical characteristics of the study groups—RRMS patients and healthy controls—are given in Table 1. There were no differences between the RRMS patients and the control group in terms of age, sex structure, and mean BMI value. In the RRMS group, the mean EDSS score was 2.52 ± 1.34, and the mean duration of the disease was 11.21 ± 5.57 yrs. The most frequently used DMT was dimethyl fumarate (*n* = 49), followed by interferon β (*n* = 29), glatiramer acetate (*n* = 17), fingolimod (*n* = 8), teriflunomide (*n* = 8), and natalizumab (*n* = 4).

**Figure 1:**
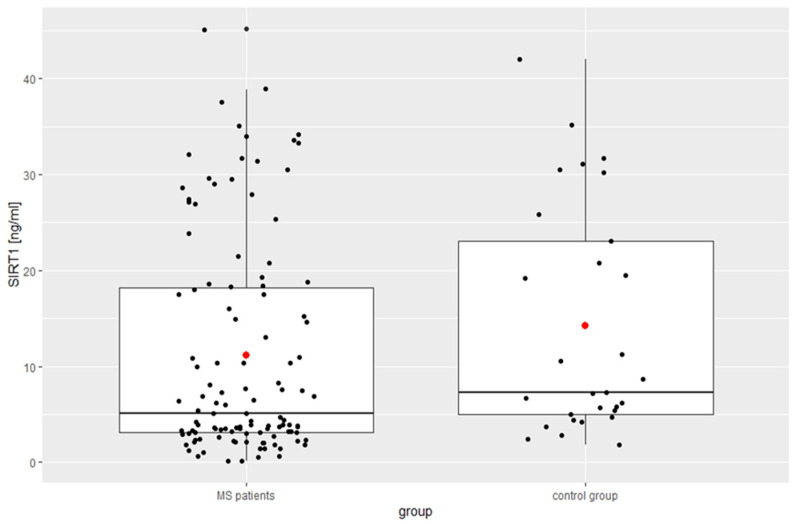
Concentration of SIRT 1 in the study group.

**Figure 2:**
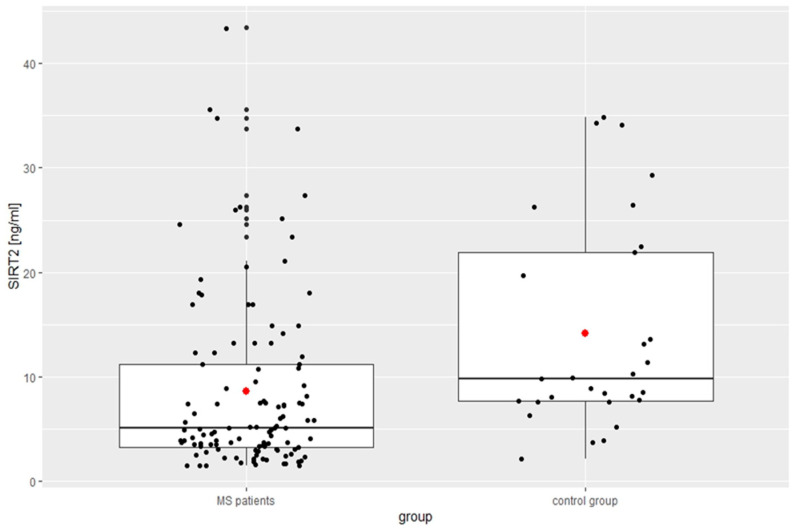
Concentration of SIRT 2 in the study group.

The mean fatigue value based on the results of the MFSI scale for the study group was 40.84 ± 19.27, which was significantly higher than that of the control group. The results of FSS indicated fatigue in 58 patients (50.4%). The mean scores of the RRMS patients were significantly higher than those of the controls. Based on the ISI score, 49.6% (*n* = 57) indicated no clinically significant insomnia; 30.4% (*n* = 35), sub-threshold insomnia; 17.4% (*n* = 20), clinical insomnia of moderate severity; and 2.6% (*n* = 3), severe clinical insomnia. According to the AIS results, insomnia occurred in 47 (41%) MS patients and in 8 (20.5%) of the controls, with no significant differences between the groups. In 106 (92%) patients of the RRMS group, the result of SDMT was below the age-adjusted norm, and its mean value was significantly lower than in the controls. The results of the modified Low questionnaire score in the patient group was significantly higher than in the control group (5.23 ± 4.54 points vs. 2.86 ± 2.33, *p* = 0.03). The score range was between 0 and 17; the median value was equal to 4 points. Dysautonomia was found in 89 patients (77%): 1–3 points (unspecified dysautonomia) in 25 patients, 4–7 points (mild dysautonomia) in 29 patients, 8–11 (moderate dysautonomia) in 20 patients, and severe dysautonomia (>12 points) in 15 patients. According to the HADS results, depressive symptoms were present in 66 RRMS patients (57%) and anxiety in 55 (48%). The HADS mean values obtained in the present study for people with MS was higher (anxiety: 7.75 ± 4.24 vs. 7.10 ± 3.70 and depression: 8.09 ± 4.30 vs. 6.59 ± 4.29) but without statistical difference compared to the control group (Table 1). Table 2 and Figure 1 and Figure 2 show the mean serum concentrations of SIRT1 and SIRT 2; both were of a significantly lower level in comparison with the controls.

In the study group, significant difference was found between the sexes in the level of SIRT1 and SIRT 2, which was statistically higher in the men than the women (SIRT1 15.7 ng/mg vs. 9.0 ng/mL, *p* = 0.002; SIRT2 11.3 ng/mL vs. 7.3 ng/mL). Other results were observed in the controls: the level of SIRT1 was higher in the women than the men (16.7 ng/mL vs. 8.7 ng/mL; *p* = 0.027), while no differences were found between the sexes in the level of SIRT2 (15.7 ng/mL vs. 10.9 ng/mL, *p* = 0.27). No significant correlations with age were noted for the level of SIRTs in the study group.

Among the MS-related variables and the results of the performed tests, the only significant positive correlation with the SIRT concentration was found for EDSS score (Table 3). No other significant relationships were revealed.

## 4. Discussion

Our study focused on evaluating the level of SIRT1 and SIRT2 in the serum of patients with MS because of their activity profile and previous evidence indicating their potential role in MS pathology [2,19]. Both SIRTs have been shown to mediate the differentiation of mesenchymal stem cells or progenitor cells into neurons and oligodendrocytes. SIRT2 contributes to the formation and interactions of the myelin sheath, as well as to the arborization of axons [20,21]. Moreover, SIRT 1 and 2 are also involved in numerous signaling pathways (e.g., the nuclear factor/NF-κB p65 pathway and forkhead box protein O (FOXO) 1/3 activation), which are associated with a modulating inflammatory response in the CNS environment (with a particular role of microglia) and with preventing neurodegeneration (via inhibition of apoptosis and oxidative stress-related injury) [2,22]. In experimental models of MS (i.e., experimental autoimmune encephalomyelitis—EAE), the activation of SIRT1 was shown to reduce neuronal injury in brain, spinal cord, and retinal ganglion cells, preventing subsequent neurological deficit. [23]. A low level of SIRT1 was associated with enhanced activation of T-cells and increased production of proinflammatory cytokines [24]. A decreased level of SIRT 2 was shown to not only promote neuroinflammation but also to delay the formation and repair of myelin [25,26,27,28]. Following these findings, the associations of SIRTs with targeted therapeutic interventions have been also investigated in animal models of demyelination. Resveratrol (SIRT1 activator), modulators of the AMPK/SIRT1 signaling pathway, and SIRT2 activators appeared to attenuate immune-mediated neuronal damage and axonal loss and induce maturation of oligodendrocytes, as well as to ameliorate clinical/behavioral symptoms [4,29,30]. These data indicate an important role of SIRTs in the pathogenesis and development of MS.

However, contradictory findings from a few studies [31,32] indicated that the inhibition of SIRT1 diminishes Th17 cells’ pro-inflammatory activity and clinical symptoms of EAE, while the locally increased expression of SIRT1 may prevent the proliferation of astrocytes and remyelination processes. We found that the serum levels of SIRT1 and SIRT2 (with especially high significance for the latter) were lower in the patients with RRMS in comparison with the healthy controls, which seems to correspond with the increased neuroinflammatory processes in the course of the disease. Other clinical studies in this field mainly concerned SIRT1 and showed somehow divergent results.

In a similar manner to our findings, a reduced level of SIRT1 was demonstrated in the serum and peripheral blood mononuclear cells (PBMCs) of the MS patients [10,33], parallel to a decrease in SIRT1 gene expression [34]. The study by Kubiliute et al. [35] confirmed links between SIRT1 gene polymorphism and the development of optic neuritis in the course of MS but did not reveal differences in SIRT 1 blood levels between the MS patients and controls. In turn, Pennisi et al. [36] found significantly higher plasma levels of SIRT1 in MS patients in comparison with healthy controls and associated this with the changes in redox status observed in MS. Interesting prospective studies [9,37] conducted in RRMS patients demonstrated a decrease in SIRT1 mRNA and phosphorylated SIRT1 expression in PBMCs during relapses, when compared to remissions. The only available clinical study on SIRT2 showed that level of Ig anti-SIRT2 in the cerebrospinal fluid of MS patients was higher than in the controls [38]. The discrepancies between the cited studies seem to be associated with methodological differences. However, the majority of the authors suggested that SIRT1 might be considered as a marker for the disease activity. We conducted an evaluation of the SIRT level in the RRMS patients during a remission, having excluded recent relapses as potentially confounding factors. Furthermore, all the patients were being treated with DMT, which is supposed to have a long-term impact on the disease activity. In a few reports, levels of SIRT 1 were specifically related to DMT. MS patients treated with interferon-β showed reduced expression of SIRT 1 in saliva compared to the controls, while no such differences were found for those treated with fingolimod [39]. Another study [9] showed the difference in phosphorylated SIRT 1 expression between responders and non-responders to glatiramer acetate, with the higher level in the former. Due to the variety of DMT used in the study group and differences in the duration of treatment, we abstained from including DMT as a variable in the analysis. However, links between SIRT level and the response to DMT seem an interesting direction of further investigation.

In our study, the level of SIRT1 was higher in the women than in the men in the control group, while the opposite was found in the RRMS patients. Previous studies have indeed demonstrated sex differences in levels of SIRTs in healthy persons [40,41]. Healthy women, especially before menopause, tend to have 30–40% higher levels of SIRT1 compared to men [40,42,43]. These differences are probably influenced by hormonal factors, particularly estrogen, which has been shown to upregulate the expression of certain sirtuins (mostly SIRT1). Sasaki et al. highlighted the role of estrogen in SIRT1-associated vascular senescence and atherosclerosis, suggesting that higher SIRT1 levels in women before menopause reflect protective effects against metabolic and cardiovascular diseases [44]. Similar sex differences were observed in the studies on links between SIRT1 and metabolic rate and the effects of caloric restriction in healthy persons and were also attributed to the influence of sex hormones upon the response to metabolic alterations [43,45]. With regard to pathologic conditions, a greater preponderance of autoimmune inflammatory processes in women should be also considered as the factor contributing to sex differences in the SIRT level. Furthermore, a reduction in the level of sirtuins, especially SIRT1, in neurodegenerative diseases (such as Parkinson’s disease or Alzheimer’s disease) was also more commonly observed in women than in men [46,47,48].

In our study groups (both in the MS patients and controls), ca 80% of the women were in the premenopausal period. However, we did not consider their hormonal status (including, e.g., the use of oral contraceptives or hormonal replacement therapy) as an additional variable in the analysis. These potential relationships between sex and SIRT levels, mediated by hormonal activity, seem interesting areas for further exploration.

With regard to MS-related variables, a significant correlation was found between the level of both SIRT1 and 2 and the EDSS score (which indicated mild to moderate disability). Ciriello et al. also demonstrated such a correlation for p-SIRT1 (phosphorylated SIRT1) expression, but only for the subgroup of patients with mild disability (EDSS) [9]. Batoee et al., in turn, observed an increase in SIRT1 gene expression along with the decrease in EDSS score in MS patients treated with a statin, but without a significant correlation between these variables [49]. Apart from an overall measure of MS-related disability, we aimed to evaluate its particular aspects, associated with advanced functions of CNS, considering the suggested role of SIRT1 and SIRT2 in underlying processes. In animal models, SIRT1 was demonstrated to contribute to the plasticity of synapses and the branching of neuronal networks, the background for processes of memory and learning [19,50]. Metabolic pathways engaging SIRT1 affect the function of the hypothalamic–pituitary axis, which is associated with neuroendocrine, autonomic, and mood regulation. Furthermore, SIRT1 was found to modulate the expression of “circadian clock” genes, thus regulating the sleep–wake cycle [51,52,53,54]. These findings suggest a relevant role of SIRT1 in the background for information processing and the adaptive reaction to stress, as well as the development of depression and anxiety.

In view of that, we undertook an investigation of SIRT levels with regard to “invisible disability”, constituting a substantial burden to MS patients and including cognitive impairment, autonomic dysfunction, sleep disturbances, mood disorders, and fatigue. For the assessment of these issues, we applied the self-assessment tools, which are validated and dedicated for MS patients. Indeed, fatigue was reported by almost half of the study group and clearly differentiated them from the healthy controls. Similar results were found for impaired performance in the domains of attention, processing speed, and executive functions (measured by SDMT) and for the clinical symptoms of dysautonomia. Surprisingly, despite the relatively high frequency of insomnia, depression, and anxiety in the MS group, it did not differ significantly from the results in the controls (which otherwise highlights the social importance of these problems). However, we did not reveal a significant relationship with the level of SIRT1 or SIRT2 for any of the above measures. There are only a few clinical studies on SIRTs (and none concerning MS) available to confront our findings. In the patients with major depressive disorder, the blood expression of SIRT1 was lower than in the healthy controls or in those effectively treated [55,56]. On the other hand, SIRT2 expression did not correlate with cognitive performance evaluated in a therapeutic trial targeted at amyloid precursor protein in Alzheimer’s disease [57]. Supposedly the putative links between SIRTs and investigated CNS dysfunctions are more complex, and an interplay of many contributing factors should be considered.

We believe that the strength of this study is associated with an attempt to investigate the level of SIRT1 and SIRT2 in a representative group of MS patients. We focused on RRMS as the most common type of disease, aiming at the relative homogeneity of the group (despite differences in disease duration and the use of DMT). Furthermore, our goal was to evaluate the SIRT level with regard to a wide panel of clinical measures reflecting multidimensional aspects of MS-related disability, particularly the “invisible” ones (including cognition, fatigue, sleep, and mood disturbances)—which to our knowledge has not been undertaken so far. Obviously, the limitations of the study have to be taken into account while interpreting its results. Excluding progressive forms of MS from the study group (for the reasons outlined above) might impede the generalizability of findings. The SIRT level was measured only in serum (because of its easier availability and thus a chance for greater sample size) and not in CSF, which would probably better reflect the SIRTs’ role in the CNS environment. Finally, the cross-sectional mode of study could not provide the insight into the dynamics of the SIRT levels in the course of disease. Considering these limitations (as well as the partly contradictory findings from other reports), further studies in this field seem feasible. They should comprise large and well-defined groups of MS subjects and involve longitudinal assessment of the SIRT level in body fluids during long-term observation, aimed at capturing the dynamics of the disease in its different stages. The relationships between SIRT levels and clinical and radiological disease outcomes should be also analyzed, considering the natural course of MS, the response to treatment, and other contributing factors (e.g., aging, hormonal activity, and comorbidities). The potential of SIRTs as monitoring and prognostic biomarkers for MS activity and/or progression, as well as novel therapeutic targets, apparently deserves further exploration.

## 5. Conclusions

The serum level of SIRT1 and SIRT2 was decreased in the patients with RRMS (especially in the female ones) and correlated with the degree of neurological deficit. No other relationships were found between the SIRT level and particular aspects of MS-related disability (autonomic, cognitive, and behavioral dysfunction). The role of SIRTs as biomarkers of MS activity or therapeutic response warrants further investigation.

## Figures and Tables

**Table 1:** Characteristics of the groups.

	Study Group *n* = 115	Control Group *n* = 39	*p*-Value
Age [years]	43 ± 9.91	42.5 ± 10.65	0.76
Sex [F/M]	78/37	27/12	
BMI	24.5 ± 4.25	25.98 ± 5.09	0.09
AIS	6.71 ± 4.35	6.00 ± 4.01	0.41
ISI	8.45 ± 6.44	6.62 ± 5.71	0.16
Low questionnaire	5.23 ± 4.54	2.86 ± 2.33	0.03
HADS—depression	8.09 ± 4.30	6.59 ± 4.29	0.10
HADS—anxiety	7.75 ± 4.24	7.10 ± 3.70	0.44
SDMT	38.71 ± 12.43	55.14 ± 8.88	<0.05
FSS	35.97 ± 14.70	23.60 ± 12.13	<0.05
MFSI	40.84 ± 19.27	22.83 ± 14.96	<0.05

Data expressed as mean ± SD. Abbreviations: BMI, body mass index; AIS, Athens Insomnia Scale; ISI, the Insomnia Severity Index; HADS, the Hospital Anxiety and Depression Scale; SDMT, the Symbol Digit Modalities Test; FSS, Fatigue Severity Scale; MFSI, Modified Fatigue Impact Scale.

**Table 2:** Levels of sirtuins in groups.

	Study Group	Control Group	*p*-Value
SIRT1 [ng/mL]	11.14 ± 11.39	14.23 ± 12.00	0.04
SIRT2 [ng/mL]	8.62 ± 8.39	14.20 ± 10.03	<0.01

**Table 3:** Correlations between concentration of SIRTs and clinical variables in the study group.

	SIRT 1 [ng/mL]		SIRT 2 [ng/mL]	
	R	*p*	R	*p*
Disease duration (years)	−0.055	0.878	−0.003	0.626
EDSS	−0.253	0.018	−0.143	0.016
BMI	0.151	0.137	−0.147	0.218
AIS	−0.130	0.220	0.143	0.094
ISI	−0.068	0.456	−0.086	0.708
Low questionnaire	−0.090	0.324	−0.011	0.121
HADS—depression	0.061	0.814	−0.140	0.473
HADS—anxiety	−0.076	0.422	0.018	0.451
SDMT	0.004	0.559	−0.050	0.590
FSS	−0.103	0.175	−0.020	0.518
MFSI	−0.149	0.059	−0.109	0.208

Abbreviations: BMI, body mass index; EDSS, Expanded Disability Status Scale; AIS, Athens Insomnia Scale; ISI, the Insomnia Severity Index; HADS, the Hospital Anxiety and Depression Scale; SDMT, the Symbol Digit Modalities Test; FSS, Fatigue Severity Scale; MFSI, Modified Fatigue Impact Scale.

## Data Availability

The original contributions presented in the study are included in the article; further inquiries can be directed to the corresponding authors.
